# Transwoman Elite Athletes: Their Extra Percentage Relative to Female Physiology

**DOI:** 10.3390/ijerph19159103

**Published:** 2022-07-26

**Authors:** Alison K. Heather

**Affiliations:** Department of Physiology, School of Biomedical Sciences, University of Otago, Dunedin 9054, New Zealand; alison.heather@otago.ac.nz

**Keywords:** sex differences, exercise physiology, transgender, testosterone, hormone therapy

## Abstract

There is increasing debate as to whether transwoman athletes should be included in the elite female competition. Most elite sports are divided into male and female divisions because of the greater athletic performance displayed by males. Without the sex division, females would have little chance of winning because males are faster, stronger, and have greater endurance capacity. Male physiology underpins their better athletic performance including increased muscle mass and strength, stronger bones, different skeletal structure, better adapted cardiorespiratory systems, and early developmental effects on brain networks that wires males to be inherently more competitive and aggressive. Testosterone secreted before birth, postnatally, and then after puberty is the major factor that drives these physiological sex differences, and as adults, testosterone levels are ten to fifteen times higher in males than females. The non-overlapping ranges of testosterone between the sexes has led sports regulators, such as the International Olympic Committee, to use 10 nmol/L testosterone as a sole physiological parameter to divide the male and female sporting divisions. Using testosterone levels as a basis for separating female and male elite athletes is arguably flawed. Male physiology cannot be reformatted by estrogen therapy in transwoman athletes because testosterone has driven permanent effects through early life exposure. This descriptive critical review discusses the inherent male physiological advantages that lead to superior athletic performance and then addresses how estrogen therapy fails to create a female-like physiology in the male. Ultimately, the former male physiology of transwoman athletes provides them with a physiological advantage over the cis-female athlete.

## 1. Introduction

Sports have been a major part of human culture throughout history, with ancient Greeks and Romans showing mastery through competition. Indeed, few areas of culture have the power to influence people as strongly as sports. It is often the intense emotional bond fans develop with teams and players that underlies their passion for sport. Fans can live vicariously through their chosen elite athletes and sportsmen and women are often considered role models for society. Amateur athletes and young emerging hopefuls strive to reach the highest levels within their chosen sport by modeling their idols.

Given the passion associated with sports, any controversy surrounding a result can lead to strong debate, especially if there is a perception that fairness has been jeopardized. The concept of fairness is the justification for dividing most sports into male and female categories. Without such segregation, females would have little chance of winning. World records show that males consistently perform better in sports [1,2,3,4,5]; the most recent Olympic gold, silver, and bronze medal results show that females would not have medaled in an open competition for a wide variety of sports (same distance raced by male and female) (Table 1; https://www.olympic.org/olympic-results (accessed on 1 January 2019). This remains despite increased female participation in sports [6] and female-centric technologies applied to training and equipment. Despite the recognition of the male dominance of athletic performance, the male and female divisions are becoming blurred as transgender women (individuals that are born as male and identify as female) athletes enter the female division. The inclusion of transgender women athletes has raised concerns about the fairness of the female division for biological female athletes (ciswomen, where gender identity aligns with their biological sex).

To compete in the female division as defined by current International Olympic Committee (IOC) regulations, a transgender woman athlete must agree to gender-affirming hormone therapy to lower, and then maintain, testosterone levels to below 10 nmol/L for one year (IOC Consensus Meeting on Sex Reassignment and Hyperandrogenism November 2015). Estradiol therapy is the common hormonal regime used to reduce testosterone levels in transwomen athletes [7,8].

The underlying question asked in this descriptive review is whether the IOC 2015 guidelines that allow transgender women to compete in the female division are fair. In order to address this question, the review aims to (A) explain the physiology behind why males perform better in sport and (B) document why male physiology cannot simply be reformatted into female physiology by estrogen therapy in transwomen athletes. In preparing this review, studies targeting (1) sex differences in athletic performance (2) testosterone administration to males or females and how this affects athletic performance and (3) transfemale or transmale physiology post-transitioning were included. The objective of this review is to increase awareness of male physiology and how it pertains to athletic performance so as to highlight issues facing the female sports division. Most importantly, the review aims to identify key physiological factors that need to be considered by sporting organizations and relevant authorities when interpreting fairness in sport.

## 2. Male Physiology Provides an Athletic Performance Advantage

**Figure 1:**
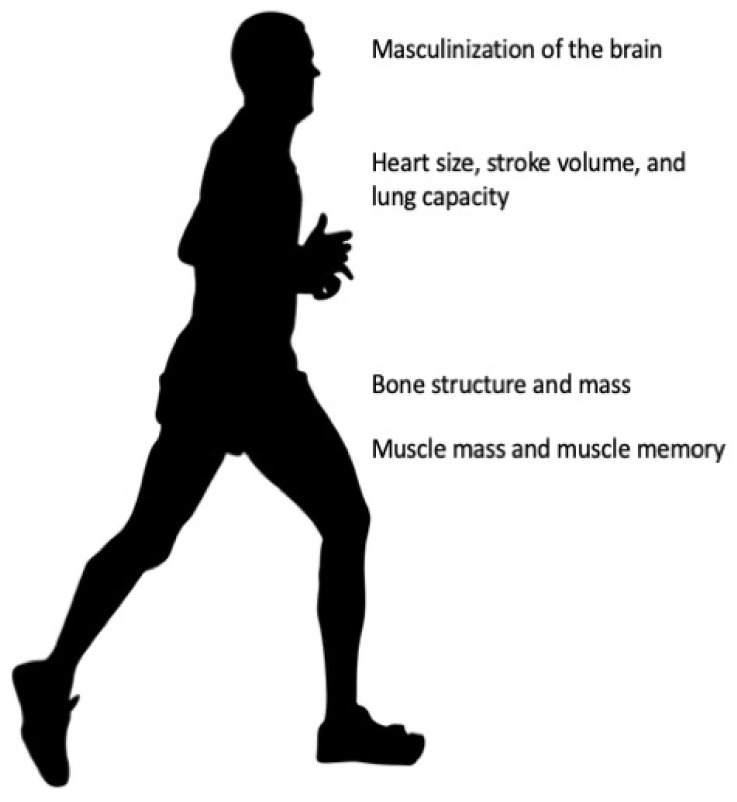
Irreversible changes to male physiology. Figure 1 shows the irreversible changes of testosterone conditioning throughout life. Testosterone masculinizes the brain in utero and during early life. Testosterone drives anatomical structure design specific to the male skeleton. Testosterone drives muscle mass, muscle fiber type, and muscle memory. Most of the effects driven by testosterone cannot be reversed with estradiol (or cross) hormone therapy.

Testosterone drives anatomical and physiological sex differences in the human body (Figure 1). These sex differences can be architectural and therefore permanent, or can be influenced by adult-level, circulating testosterone concentrations, and therefore modifiable. Permanent sex differences that affect athletic performance involve the (i) brain, (ii) skeletal structure, and (iii) cardiorespiratory system. Modifiable sex differences include testosterone effects on (i) muscle mass and strength and (ii) aerobic capacity.

### 2.1. Prenatal Testosterone and the Male-like Brain

Anatomical sex differences in the brain appear during early life when the in utero and postnatal surges of testosterone masculinize the male brain [9,10]. Ascribing specific behaviors to these anatomical sex differences has been challenging [11] and may involve differences in cell morphology as well as the connections between brain networks [12,13,14,15,16]. For example, diffusion magnetic resonance imaging (MRI) shows increased intraconnectivity in males for regions of the brain attributed to perception-action coordination, auditory/visual spatial awareness and processing, cognitive processes and complex reasoning and control [16]. Females, on the other hand, show more interconnectivity in those regions of the brain attributed to memory, social cognition, and non-verbal reasoning. The sex-specific connectivity of these subnetworks may underlie the ability of males to show consistently higher levels of motor and visual spatial skills in addition to elevated sensory input from vision and proprioception [17,18,19,20].

There is little debate that better motor skills, visual spatial skills, and proprioception will improve coordination and subsequent athletic performance. These differences in brain structure are evident in childhood, with 8–13 year olds showing behavior patterns that become further differentiated with the onset of puberty and aging into adulthood [16].

An aggressive, competitive nature also underpins better athletic performance [21]. Although it is difficult to attribute prenatal testosterone exposure directly to levels of aggression in the adult, indirect evidence suggests that such a relationship may exist. Development of the fourth digit (4D), but not the second digit (2D), is highly sensitive to testosterone, so that in utero androgen exposure results in lower 2D:4D ratios in males compared to females and is considered an index of prenatal testosterone exposure [22,23,24,25]. There is a clear association between 2D:4D ratios and male-typical behaviors [24,26] and, interestingly, professional male football players with low 2D:4D ratios receive more yellow or red card penalties [27]. Even with females, lower 2D:4D ratios in females are associated with the more aggressive form of sabre fencing [28]. Such examples suggest the possibility that in utero androgen exposure leads to later-life aggressiveness.

### 2.2. Testosterone and Muscle Mass

For many decades, it has been recognized that testosterone drives muscle mass and clear sex differences exist. For example, elite male athletes have, on average, more muscle mass than elite female athletes, for any given body weight [29]. Males have approximately twice the cross sectional area of upper body muscle, and 30% more cross sectional area of lower body muscle relative to females [30]. The difference in muscle mass emerges during puberty as circulating testosterone levels increase in boys [31].

Circulating testosterone is the key physiological parameter driving muscle mass in both males and females [32,33,34]. For example, 19–40 year old males given supraphysiologic doses of testosterone for 10 weeks increased muscle mass, muscle size, and strength by up to 10%. If this treatment was combined with strength training, the increase in muscle mass was 27% [32]. A follow-up study by the same laboratory [33] showed that the testosterone effect on muscle mass was dose-dependent. Males, 18–35 years old, were treated with a gonadotropin-releasing hormone (GnRH) agonist to suppress endogenous testosterone production and then administered testosterone for 20 weeks at either 25, 50, 125, 300, or 600 mg, resulting in circulating concentrations of 8.8, 10.6, 18.8, 46.7, and 82.2 nmol/L, respectively, covering low physiological to supra-physiological levels. Supraphysiological concentrations (>40 nmol/L) increased leg muscle mass and strength, whereas mid-range testosterone level administration increased muscle mass with maintenance of leg strength. Interestingly, low testosterone levels (<10 nmol/L) maintained leg muscle mass and strength. This latter finding is in keeping with research in rodents that shows testosterone is not needed for muscle maintenance in adult male mice [35]. In this study, orchidectomised mice aged from young through to old were assessed for muscle mass after 28 days of testosterone depletion. Only the youngest mice showed reduced muscle mass, while the older, adult mice showed no effect on muscle mass. Therefore, it appears that testosterone may not be critical for maintenance of muscle mass in mature male mice [35]. In females, testosterone administration to raise circulating levels from 0.9 nmol/L to 4.3 nmol/L in young women (average 28 years) increased muscle mass and strength, as well as enhanced athletic performance as evidenced by time to exhaustion and Wingate testing [36].

### 2.3. Testosterone and Bone Structure

Bone structure and bone length changes in both sexes as children progress through puberty, with estradiol and testosterone having important roles in bone growth [37,38]. However, the effects of testosterone are stronger than those of estradiol, as exemplified by the 10% greater bone mass density and the larger and longer bones in post-pubertal males. These sex differences in bone structure provide males with increased fulcrum power, improving jumping, throwing, and other movements requiring explosive actions. The stronger bones also tolerate more trauma and thus males are more resistant to injury. Larger bones in males provide a greater articular surface that, in turn, allows placement of more skeletal muscle. For example, broader shoulders in males allows the build-up of more muscle, thereby increasing upper body strength [39].

Sex differences in bone shape driven by early life testosterone exposure can affect athletic performance. The most obvious structural difference between males and females is pelvis width, with estradiol driving the wider shape required for childbirth [40]. A narrower pelvis has a direct impact on the Q angle at the knee joint. The Q angle forms between the quadricep muscles and the patellar tendon and is responsible for generating force during a knee extension. The smaller Q angle of males generates a greater force upon extension [41]. This has implications for sports that involve standing from a squatting position, kicking a ball, or a pedaling motion. There is also a sex difference in the angle formed between the humerus and ulna at the elbow, with the angle smaller in a male [42]. The smaller angle for males again allows a greater force upon extension, benefiting sports involving throwing and hitting with bats and rackets.

### 2.4. Testosterone and the Cardiorespiratory System

Early life testosterone exposure also drives sex differences in the cardiorespiratory system. Females have, on average, a 10–12% smaller lung volume than males, accounting for height, age, and within sex variation [43]. This smaller lung volume is established within the first few years of life in females due to a lower rate of alveolar multiplication [44,45] and shorter diaphragm that reduces ribcage dimensions [46]. These anatomical differences therefore drive a lower oxygen uptake capacity in females [43,44]. Females also have a heart size that is about 85% that of males, relative to body size [47]. This anatomical difference decreases the volume of blood that can be pumped to the body with every contraction of the heart. The larger heart size of males translates to a larger left ventricle and therefore, stroke volume. On average, the stroke volume of females is just one-third that of males. As such, a female’s heart rate must increase proportionally more to achieve a cardiac output necessary to supply active skeletal muscle with enough oxygen.

Active skeletal muscles require the efficient delivery of oxygen, and aerobic capacity is essential for athletic performance. Males have much higher arterial oxygen levels, primarily due to testosterone-regulated synthesis of hemoglobin [48]. The increased hemoglobin levels coupled with increased lung capacity provides males with a distinct respiratory and oxygen carrying advantage over females. It is consistently reported that hemoglobin concentrations are 12% higher in males than females, and this sex difference in hemoglobin emerges during puberty driven by testosterone [48]. Administration studies show that testosterone increases hemoglobin levels in a dose-dependent fashion [49], and medical castration to lower circulating testosterone levels in prostate cancer patients decreases hemoglobin levels [50].

These effects of testosterone on oxygen carrying capacity, together with the anatomical advantages of male anatomy, help explain the superiority of the male cardiovascular system as it relates to athletic performance. The VO_2 max_ of an elite male is in the order of 70–85 mL/kg/min, while that of females is 60–75 mL/kg/min; a difference of 15–30% [51].

## 3. Male Physiology Cannot Be Reformatted into Female Physiology by Estrogen Therapy

As noted above, the combined effect of testosterone-driven male physiology culminates in greater athletic performance, as evidenced by the dominance of males with respect to World Records and Olympic Gold medals [1,2,3,4,5]. In strength-related sports, world records can differ by 10–30% between males and females [1,2,3,4,5]. As such, to ensure a fair playing field in the female division, a transgender woman athlete should not retain any advantage from her prior testosterone-driven physiology. The evidence below indicates that male physiology cannot be reformatted to female physiology simply by gender-affirming estradiol therapy in a transgender woman training at a high-performance level.

### 3.1. Difficulties in Achieving Female Levels of Circulating Testosterone in Estrogen-Treated Transwomen

The estrogen treatment regimens used in transgender women aim to lower testosterone levels to within the female range (<1 nmol/L) [52]. However, hormone therapy alone has met limited success in suppressing testosterone levels, with many transgender women failing to achieve the desired level. In recent studies of transgender women, one quartile failed to achieve any significant suppression [53] and one-third failed to suppress testosterone levels despite achieving desired estradiol levels [54]. Another study reported that only 49% of transgender women showed suppressed testosterone concentrations after 6 months or more of estrogen with the addition of antiandrogen therapy [55]. Notably, Jarin and colleagues show that testosterone levels in transgender women decreased significantly from former male levels, however nearly all participants maintained their testosterone levels above the female range [56]. Whether elite transwoman athletes experience the same difficulties in suppressing testosterone levels with estrogen therapy has not been reported.

### 3.2. Estrogen Therapy Does Not Reformat Male-Like Brain Networks

At present, there is little understanding about changes in brain structure and function in transgender individuals. Almost all research in this area has focused on trying to delineate neurobiological pathways that underpin transgenderism, rather on specific areas of the brain related to athletic performance. Overall, current evidence indicates that transgender hormone therapy either has no effect or generates structural and functional changes in the brain that are intermediate between biological males and females [57,58,59].

For transgender women, gender-affirming surgery and estrogen therapy have typically, but not always [57], been shown to reduce brain size and increase ventricular volume towards the parameters measured for biological females [60,61,62,63,64]. While MRI has shown consistent sex differences in functional connectivity within the brain, any alterations following hormonal treatment in transgender individuals remain unclear with studies typically showing no change or the appearance of an intermediate male–female state [63,64,65,66,67]. Notably, the biological male dominance in spatial ability, visual memory tasks, and perception [65,66,67] shows no decrease in transwomen after 12 months of estrogen therapy [68]. A meta-analysis of transgender individuals receiving gender affirming hormone therapy for 3–12 months shows transgender men had a significant enhancement in visuospatial ability (ref. [69]), with no significant changes for either transgender men or women in verbal memory, verbal reasoning, verbal working memory, computation, or motor coordination. There is an estrogen therapy-related change in transgender women if treated for longer than 12 months with a decreased proneness towards anger and aggressiveness [70].

While more research is required for definitive answers, the current data demonstrates that estrogen therapy in transwomen can drive some brain structures towards that of the biological female [71,72,73] but does not do so quickly and appears to not reformat the sex-typical male brain responses into a female-like brain. This is compatible with the concept that both the in utero and perinatal surges of testosterone secretion occurring in males drive permanent sex differences in brain structure and function which are apparent in young boys before puberty [74]. Human spatial ability, for example, appears beneficially affected by prenatal androgens [75,76], with second trimester testosterone levels correlated positively in biological females and negatively in biological males at age 7 [77].

### 3.3. Estrogen Therapy Does Not Reformat Male Skeletal Architecture but Does Decrease Muscle Mass

As there is a dose-dependent relationship between testosterone and muscle mass [32,33,34], lowering circulating testosterone levels from an average male level to <10 nmol/L should decrease muscle mass in a transwoman. Indeed, studies report muscle mass loss, with a 5% loss for lower limb mass or a 9.4% for total muscle mass loss after 12 months of estrogen therapy [78,79]. The loss of muscle mass did not, however, associate with loss of strength or muscle fiber density or performance [79]. For example, prior to transitioning, transwoman airforce personnel recorded a 12% faster time for a 1.5 mile run than their biological female peers that declined to a 9% difference after 2–2.5 years on estrogen therapy [80]. This decline in performance is similar to a self-reported study of running times in transwomen in the 12 months after transitioning [81]. While such results represent a marked decrease in performance, the running times of the transwomen group remain significantly higher than those of the biological women [80], despite prolonged estrogen therapy. The performance benefit of prior testosterone exposure for the running test is likely attributable to not only muscle mass but male skeletal architecture that, as discussed earlier includes longer limbs, a narrower pelvic structure and a greater cardiorespiratory size—all of which will not respond to changes in circulating testosterone levels in adulthood. Further to this, studies show that there is no bone mass loss in transwomen after 28–63 months of estrogen therapy [82].

As discussed earlier, sex differences in muscle mass in elite athletes can be 50–75% [30] in favor of males, thus the decrease of 5–10% reported in studies of transwomen after 1–2 years of estrogen therapy will most likely provide, at most, a modest reformatting of male muscle strength in the transwoman athlete. Further evidence to support maintenance of muscle mass in the face of lowered testosterone levels is observed in prostate cancer patients. Such patients are on androgen-deprivation therapy for extended periods to lower testosterone levels to very low levels (i.e., within the female range) but only report a small loss (2–4%) in muscle mass over 12 months [83]. Notably, this effect can be mitigated with an exercise training program [79,84,85]. Therefore, it follows that a transwoman athlete following a high-performance training program enabling competitiveness at an elite level throughout the 12-month estrogen therapy transition period could similarly mitigate muscle mass loss.

The difficulty of reformatting muscle physiology to female levels in transwomen likely results from their life-long exposure to testosterone prior to transitioning and prior levels of exercise as a male. Increased muscle mass arises from hypertrophy of individual muscle fibers in response to enhanced protein synthesis within the cells. Muscle cells recruit nuclei from nearly helper satellite cells to increase protein synthesis and allow muscle cell hypertrophy [86,87,88,89]. The most important stimulus for muscle hypertrophy is exercise and thus, muscles subjected to overload exercise recruit nuclei. If the muscle then undergoes a period of rest, the muscle cell will atrophy back to its baseline size but will retain the nuclei acquired during the hypertrophic phase. In humans, nuclei numbers are very stable and may even be permanent [90]. When these cells with higher numbers of nuclei are again subjected to overload exercise, the cells can quickly ramp up protein synthesis and hypertrophy. Thus, muscle cell nuclei number represents a form of “muscle memory” that is a functionally important indicator of prior strength and explains how previous exercise improves the ability to regain muscle mass later in life, even after long sedentary periods [91,92].

Mouse studies have shown that testosterone administration for 2 weeks significantly increased muscle cell hypertrophy and generated an increase in muscle cell nuclei number that was maintained for the next 8 weeks. Mice were then subjected to overload training and those animals that had been treated with testosterone showed a 3-fold increase in muscle hypertrophy compared to controls [88].

Muscle memory is, therefore, a critical factor when considering the physiology of transwoman athletes. Prior to transitioning, the post-pubertal exposure to high androgen levels, relative to a biological female, would increase muscle cell nuclei number [93], a long-term benefit that is not quickly, if ever, reversed. This androgen-induced muscle memory effect has been used to argue that a two-year sanction from elite competition is not sufficient for those athletes found to have consumed exogenous androgens because of the potential on-going athletic performance advantage [94,95].

### 3.4. Estrogen Therapy and Effects on the Cardiorespiratory System

The physiological parameter that will be affected most by estrogen therapy is VO_2 max_ or maximum aerobic performance. Due to the dependence of VO_2 max_ on hemoglobin levels, and the relationship between testosterone and hemoglobin, reduced testosterone will decrease VO_2 max_. Importantly, hemoglobin is sensitive to testosterone even at low concentrations [96], with dose-dependent increases measured in females within the 0–4 nmol/L range [96]. Males on androgen-deprivation therapy show a significant 9% drop in hemoglobin levels after 12 months, with a further variable decline by 12.8–29% after 24 months [97]. This decrease in hemoglobin will likely decrease maximal aerobic performance. The hemoglobin response to testosterone concentrations, whether increasing or decreasing, occurs within weeks [98], thus, if the hemoglobin levels are to remain lowered in transfemale athletes, circulating testosterone levels must remain consistently reduced. As discussed above, achieving testosterone suppression to very low levels in transwomen can be difficult [53,54,55,56], where nearly all transwomen involved in reported studies failed to achieve or maintain testosterone levels in the biological female range, which was consummate with a higher hemoglobin level than that of biological females [56].

While hemoglobin levels respond closely to circulating testosterone levels, other cardiorespiratory system parameters are unlikely to be impacted significantly by estrogen therapy. For example, sex differences in lung size and alveolar numbers, total heart size, left ventricular size, stroke volume, and subsequent cardiac output will not be changed significantly. All of these parameters are defined by anatomical structures that were programmed by early life and early pubertal exposure to testosterone.

## 4. Conclusions

Testosterone drives much of the enhanced athletic performance of males through in utero, early life, and adult exposure. Many anatomical sex differences driven by testosterone are not reversible. Hemoglobin levels and muscle mass are sensitive to adult life testosterone levels, with hemoglobin being the most responsive. Studies in transgender women, and androgen-deprivation treated cancer patients, show muscle mass is retained for many months, even years, and that co-comittant exercise mitigates muscle loss. Given that sports are currently segregated into male and female divisions because of superior male athletic performance, and that estrogen therapy will not reverse most athletic performance parameters, it follows that transgender women will enter the female division with an inherent advantage because of their prior male physiology.

The current IOC regulations allow transwomen athletes to compete if testosterone levels have been lowered to <10 nmol/L for 12 months prior to competition. While this begins to address the advantageous effects of circulating testosterone on athletic performance, it does not take into account the advantage afforded by testosterone exposure prior to transitioning. The existing data suggests that lowering testosterone to less than 10 nmol/L for 12 months decreases muscle mass but not to biological female levels and despite the decrease in mass, muscle strength can be maintained, especially if concurrently exercising. Estrogen therapy does not affect most of the anatomical structures in the biological male that provide a physiological benefit. Hemoglobin levels are lowered by estrogen therapy, and consequently, maximum aerobic effort may be lower, but this parameter will only be manifested if testosterone levels are suppressed to levels within the biological female range and maintained for extended periods of time. Reported studies show it is difficult to continuously suppress testosterone in transgender women. Given that the percentage difference between medal placings at the elite level is normally less than 1%, there must be confidence that an elite transwoman athlete retains no residual advantage from former testosterone exposure, where the inherent advantage depending on sport could be 10–30%. Current scientific evidence can not provide such assurances and thus, under abiding rulings, the inclusion of transwomen in the elite female division needs to be reconsidered for fairness to female-born athletes.

## Figures and Tables

**Table 1:** Gold medal distances or times for selected sporting events recorded for males and females at the RIO Olympics 2016.

Sport	Male	Male	Male	Female	Finishing Position for Female Gold in Male Event
**Athletics**	**Gold**	**Silver**	**Bronze**	**Gold**	
10,000 m run	27.05.17 s	27.05.64 s	27.05.26 s	29.17.45 s	32nd
100 m run	9.81 s	9.89 s	9.91 s	10.71 s	Would not have qualified for gold medal final
400 m run	43.03 s	43.76 s	43.85 s	49.44 s	Would not have qualified for the gold medal final
800 m run	1:42:15 min	1:42:61 min	1:42:93 min	1:55:28 min	Would not have qualified for the gold medal final
marathon	2.08.44 h	2.09.54 h	2.10.05 h	2.24.04 h	90th
long jump	8.38 m	8.37 m	8.29 m	7.17 m	<12th (cut off at 7.82 m)
**Triathlon**	1.45.01 h	1.45.07 h	1.45.43 h	1.56.16 h	50th
**Swimming**					
100 m freestyle	47.58 s	47.8 s	47.85 s	52.70 s	51st out of 57 in the heats
400 m individual medley	4.06.05 m	4.06.75 m	4.09.71 m	4.26.36 m	26th out of 27 in the heats
**Canoe sprint**					
K-1200 m (Kayak single)	35.197 s	35.362 s	35.662 s	39.864 s.	Would not have placed in the top 16 to make finals (cut off 38.061)

Distances are given as meters (m), and times are given as seconds (s), minutes (mins), or hours (h).

## Data Availability

Not applicable.

## References

[1] Cheuvront S.N., Carter R., DeRuisseau K.C., Moffatt R.J. (2005). Running performance differences between men and women: An update. Sports Med..

[2] Nevill A.M., Whyte G. (2005). Are there limits to running world records?. Med. Sci. Sports Exerc..

[3] Seiler S., de Koning J.J., Foster C. (2007). The fall and rise of the gender difference in elite anaerobic performance 1952–2006. Med. Sci. Sports Exerc..

[4] Berthelot G., Thibault V., Tafflet M., Escolano S., El Helou N., Jouven X., Hermine O., Toussaint J.-F. (2008). The citius end: World records progression announces the completion of a brief ultra-physiological quest. PLoS ONE.

[5] Thibault V., Guillaume M., Berthelot G., El Helou N., Schaal K., Quinquis L., Nassif H., Tafflet M., Escolano S., Hermine O. (2010). Women and Men in Sport Performance: The Gender Gap has not Evolved since 1983. J. Sports Sci. Med..

[6] Kennedy C.L. (2010). A New Frontier for Women’s Sports (Beyond Title IX). Gend. Issues.

[7] Bretherton I., Thrower E., Grossmann M., Zajac J.D., Cheung A.S. (2019). Cross-sex hormone therapy in Australia: The prescription patterns of clinicians experienced in adult transgender healthcare. Intern. Med. J..

[8] Randolph J.F. (2018). Gender-Affirming Hormone Therapy for Transgender Females. Clin. Obstet. Gynecol..

[9] Phoenix C.H., Goy R.W., Gerall A.A., Young W.C. (1959). Organizing action of prenatally administered testosterone propionate on the tissues mediating mating behavior in the female guinea pig. Endocrinology.

[10] McCarthy M.M. (2016). Sex differences in the developing brain as a source of inherent risk. Dialogues. Clin. Neurosci..

[11] de Vries G.J., Sodersten P. (2009). Sex differences in the brain: The relation between structure and function. Horm. Behav..

[12] Isgor C., Sengelaub D.R. (1998). Prenatal gonadal steroids affect adult spatial behavior, CA1 and CA3 pyramidal cell morphology in rats. Horm. Behav..

[13] Isgor C., Sengelaub D.R. (2003). Effects of neonatal gonadal steroids on adult CA3 pyramidal neuron dendritic morphology and spatial memory in rats. J. Neurobiol..

[14] Schwarz A.J., Gozzi A., Bifone A. (2008). Community structure and modularity in networks of correlated brain activity. Magn. Reson. Imaging.

[15] Tononi G., Sporns O., Edelman G.M. (1994). A measure for brain complexity: Relating functional segregation and integration in the nervous system. Proc. Natl. Acad. Sci. USA.

[16] Tunç B., Solmaz B., Parker D., Satterthwaite T.D., Elliott M.A., Calkins M.E., Ruparel K., Gur R.E., Gur R.C., Verma R. (2016). Establishing a link between sex-related differences in the structural connectome and behaviour. Philos. Trans. R Soc. Lond. B Biol. Sci..

[17] Robazza C., Bortoli L. (2007). Perceived impact of anger and anxiety on sporting performance in rugby players. Psychol. Sport Exerc..

[18] Bartlett M.L., Abrams M., Byrd M., Treankler A.S., Houston-Norton R. (2018). Advancing the Assessment of Anger in Sports: Gender Differences and STAXI-2 Normative Data for College Athletes. J. Clin. Sport Psychol..

[19] Ruiz M.C., Hanin Y. (2011). Perceived impact of anger on performance of skilled karate athletes. Psychol. Sport Exerc..

[20] Abrams M. (2010). Anger Management in Sport.

[21] Bartlett M.L., Abrams M., Anshel T.A.P.M.H., Steinfeldt J.A. (2019). Anger and aggression in sport. APA Handbooks in Psychology Series. APA Handbook of Sport and Exercise Psychology.

[22] Deaner R.O. (2013). Physiology does not explain all sex differences in running performance. Med. Sci. Sports Exerc..

[23] Deaner R.O. (2013). Distance running as an ideal domain for showing a sex difference in competitiveness. Arch. Sex. Behav..

[24] Ribeiro E., Neave N., Morais R.N., Kilduff L., Taylor S.R., Butovskaya M., Fink B., Manning J.T. (2016). Digit ratio (2D:4D), testosterone, cortisol, aggression, personality and hand-grip strength: Evidence for prenatal effects on strength. Early Hum. Dev..

[25] Kim T.B., Kim K.H. (2016). Why is digit ratio correlated to sports performance?. J. Exerc. Rehabil..

[26] Bönte W., Procher V.D., Urbig D., Voracek M. (2017). Digit Ratio (2D:4D) Predicts Self-Reported Measures of General Competitiveness, but Not Behavior in Economic Experiments. Front. Behav. Neurosci..

[27] Mailhos A., Buunk A.P., del Arca D., Tutte V. (2016). Soccer players awarded one or more red cards exhibit lower 2D:4D ratios. Aggress. Behav..

[28] Voracek M., Reimer B., Dressler S.G. (2010). Digit ratio (2D:4D) predicts sporting success among female fencers independent from physical, experience, and personality factors. Scand. J. Med. Sci. Sports.

[29] Healy M.L., Gibney J., Pentecost C., Wheeler M.J., Sonksen P.H. (2014). Endocrine profiles in 693 elite athletes in the postcompetition setting. Clin. Endocrinol..

[30] Sale D.G., Tarnopolsky M. (1999). Neuromuscular function. Gender Differences in Metabolism: Practical and Nutritional Implications.

[31] Handelsman D.J. (2017). Sex differences in athletic performance emerge coinciding with the onset of male puberty. Clin. Endocrinol..

[32] Bhasin S., Storer T.W., Berman N., Callegari C., Clevenger B., Phillips J., Bunnell T.J., Tricker R., Shirazi A., Casaburi R. (1996). The effects of supraphysiologic doses of testosterone on muscle size and strength in normal men. N. Engl. J. Med..

[33] Bhasin S., Woodhouse L.J., Casaburi R., Singh A.B., Bhasin D., Berman N., Chen X., Yarasheski K., Magliano L., Dzekov C. (2001). Testosterone dose-response relationships in healthy young men. Am. J. Physiol. Endocrinol. Metab..

[34] Hirschberg A.L. (2019). Hyperandrogenism in Female Athletes. J. Clin. Endocrinol. Metab..

[35] Davidyan A., Pathak S., Baar K., Bodine S.C. (2021). Maintenance of muscle mass in adult male mice is independent of testosterone. PLoS ONE.

[36] Hirschberg A.L., Knutsson J.E., Helge T., Godhe M., Ekblom M., Bermon S., Ekblom B. (2019). Effects of moderately increased testosterone concentration on physical performance in young women: A double blind, randomised, placebo controlled study. Br. J. Sports Med..

[37] Turner R.T., Riggs B.L., Spelsberg T.C. (1994). Skeletal effects of estrogen. Endocr. Rev..

[38] Orwoll E.S., Klein R.F. (1995). Osteoporosis in men. Endocr. Rev..

[39] Laubach L.L. (1976). Comparative muscular strength of men and women: A review of the literature. Aviat. Space Environ. Med..

[40] Huseynov A., Zollikofer C.P., Coudyzer W., Gascho D., Kellenberger C., Hinzpeter R., Ponce de León M.S. (2016). Developmental evidence for obstetric adaptation of the human female pelvis. Proc. Natl. Acad. Sci. USA.

[41] Sutherland M.A., Wassersug R.J., Rosenberg K.R. (2017). From transsexuals to transhumans in elite athletics—The implications of osteology (and other issues) in leveling the playing field. Transgender Athletes in Competitive Sport.

[42] Fornalski S., Gupta R., Lee T.Q. (2003). Anatomy and biomechanics of the elbow joint. Tech. Hand Up. Extrem. Surg..

[43] Carey M.A., Card J.W., Voltz J.W., Arbes S.J., Germolec D.R., Korach K.S., Zeldin D.C. (2007). It’s all about sex: Gender, lung development and lung disease. Trends Endocrinol. Metab..

[44] Townsend E.A., Miller V.M., Prakash Y.S. (2012). Sex Differences and Sex Steroids in Lung Health and Disease. Endocr. Rev..

[45] Thurlbeck W.M. (1982). Postnatal human lung growth. Thorax.

[46] Bellemare F., Jeanneret A., Couture J. (2003). Sex differences in thoracic dimensions and configuration. Am. J. Respir. Crit. Care Med..

[47] Leinwand L.A. (2003). Sex is a potent modifier of the cardiovascular system. J. Clin. Investig..

[48] Murphy W.G. (2014). The sex difference in haemoglobin levels in adults-mechanisms, causes, and consequences. Blood Rev..

[49] Coviello A.D., Kaplan B., Lakshman K.M., Chen T., Singh A.B., Bhasin S. (2008). Effects of graded doses of testosterone on erythropoiesis in healthy young and older men. J. Clin. Endocrinol. Metab..

[50] Grossmann M., Cheung A.S., Zajac J.D. (2013). Androgens and prostate cancer; pathogenesis and deprivation therapy. Best Pract. Res. Clin. Endocrinol. Metab..

[51] Kenney W.L., Wilmore J.H., Costill D.L. (2012). Physiology of Sport and Exercise.

[52] Gardner I.H., Safer J.D. (2013). Progress on the road to better medical care for transgender patients. Curr. Opin. Endocrinol. Diabetes Obes..

[53] Liang J., Jolly D., Chan K.J., Safer J.D. (2018). Testosterone Levels Achieved by Medically Treated Transgender Women in a United States Endocrinology Clinic Cohort. Endocr. Pract..

[54] Leinung M.C., Feustel P., Joseph J. (2018). Hormonal Treatment of Transgender Women with Oral Estradiol. Transgend. Health.

[55] SoRelle J.A., Jiao R., Gao E., Veazey J., Frame I., Quinn A.M., Day P., Pagels P., Gimpel N., Patel K. (2019). Impact of Hormone Therapy on Laboratory Values in Transgender Patients. Clin. Chem..

[56] Jarin J., Pine-Twaddell E., Trotman G., Stevens J., Conard L.A., Tefera E., Gomez-Lobo V. (2017). Cross-Sex Hormones and Metabolic Parameters in Adolescents with Gender Dysphoria. Pediatrics.

[57] Guillamon A., Junque C., Gil E.G. (2016). A Review of the Status of Brain Structure Research in Transsexualism. Arch. Sex. Behav..

[58] Kreukels B.P., Guillamon A. (2016). Neuroimaging studies in people with gender incongruence. Int. Rev. Psychiatry.

[59] Mueller S.C., Landré L., Wierckx K., T’Sjoen G. (2017). A Structural Magnetic Resonance Imaging Study in Transgender Persons on Cross-Sex Hormone Therapy. Neuroendocrinology.

[60] Seiger R., Hahn A., Hummer A., Kranz G.S., Ganger S., Woletz M., Kraus C., Sladky R., Kautzky A., Kasper S. (2016). Subcortical gray matter changes in transgender subjects after long-term cross-sex hormone administration. Psychoneuroendocrinology.

[61] Zubiaurre-Elorza L., Junque C., Gómez-Gil E., Guillamon A. (2014). Effects of cross-sex hormone treatment on cortical thickness in transsexual individuals. J. Sex. Med..

[62] Burke S.M., Manzouri A.H., Savic I. (2017). Structural connections in the brain in relation to gender identity and sexual orientation. Sci. Rep..

[63] Mueller S.C., Wierckx K., Jackson K., T’Sjoen G. (2016). Circulating androgens correlate with resting-state MRI in transgender men. Psychoneuroendocrinology.

[64] Kranz G., Hahn A., Kaufmann U., Tik M., Ganger S., Seiger R., Hummer A., Windischberger C., Kasper S., Lanzenberger R. (2018). Effects of testosterone treatment on hypothalamic neuroplasticity in female-to-male transgender individuals. Brain Struct. Funct..

[65] Linn M.C., Petersen A.C. (1985). Emergence and characterization of sex differences in spatial ability: A meta-analysis. Child. Dev..

[66] Voyer D., Voyer S., Bryden M.P. (1995). Magnitude of sex differences in spatial abilities: A meta-analysis and consideration of critical variables. Psychol. Bull..

[67] Hedges L.V., Nowell A. (1995). Sex differences in mental test scores, variability, and numbers of high-scoring individuals. Science.

[68] Miles C., Green R., Hines M. (2006). Estrogen treatment effects on cognition, memory and mood in male-to-female transsexuals. Horm. Behav..

[69] Karalexi M.A., Georgakis M.K., Dimitriou N.G., Vichos T., Katsimpris A., Petridou E.T., Papadopoulos F.C. (2020). Gender-affirming hormone treatment and cognitive function in transgender young athletes: A systematic review and meta-analysis. Psychoneuroendocrinology.

[70] Van Goozen S.H., Cohen-Kettenis P.T., Gooren L.J., Frijda N.H., Van De Poll N.E. (1995). Gender differences in behaviour: Activating effects of cross-sex hormones. Psychoneuroendocrinology.

[71] Pol H.E.H., Schnack H.G., Mandl R.C., Brans R.G., van Haren N.E., Baaré W.F., van Oel C., Collins D.L., Evans A.C., Kahn R.S. (2006). Gray and white matter density changes in monozygotic and same-sex dizygotic twins discordant for schizophrenia using voxel-based morphometry. Neuroimage.

[72] Kim T.-H., Kim G.-W., Kim S.-K., Jeong G.-W. (2016). Brain activation-based sexual orientation in female-to-male transsexuals. Int. J. Impot. Res..

[73] Kim T.H., Kim S.K., Jeong G.W. (2015). Cerebral gray matter volume variation in female-to-male transsexuals: A voxel-based morphometric study. Neuroreport.

[74] McCarthy M.M. (2016). Multifaceted origins of sex differences in the brain. Philos. Trans. R Soc. Lond. B Biol. Sci..

[75] Beking T., Geuze R., van Faassen M., Kema I., Kreukels B., Groothuis T. (2018). Prenatal and pubertal testosterone affect brain lateralization. Psychoneuroendocrinology.

[76] Nguyen T.-V., Lew J., Albaugh M.D., Botteron K.N., Hudziak J.J., Fonov V.S., Collins D.L., Ducharme S., McCracken J.T. (2017). Sex-specific associations of testosterone with prefrontal-hippocampal development and executive function. Psychoneuroendocrinology.

[77] Grimshaw G., Sitarenios G., Finegan J. (1995). Mental rotation at 7 years: Relations with prenatal testosterone levels and spatial play experiences. Brain Cogn..

[78] Elbers J.M.H., Asscheman H., Seidell J., Gooren L.J.G. (1999). Effects of sex steroid hormones on regional fat depots as assessed by magnetic resonance imaging in transsexuals. Am. J. Physiol..

[79] Wiik A., Lundberg T.R., Rullman E., Andersson D.P., Holmberg M., Mandić M., Brismar T.B., Leinhard O.D., Chanpen S., Flanagan J.N. (2019). Muscle strength, size and composition following 12 months of gender-affirming treatment in transgender individuals: Retained advantage for the transwomen. bioRxiv.

[80] Roberts T.A., Smalley J., Ahrendt D. (2020). Effect of gender affirming hormones on athletic performance in transwomen and transmen: Implications for sporting organisations and legislators. Br. J. Sports Med..

[81] Harper J. (2015). Race times for transgender athletes. J. Sport. Cult. Identities.

[82] van Kesteren P., Lips P., Gooren L.J., Asscheman H., Megens J. (1998). Long-term follow-up of bone mineral density and bone metabolism in transsexuals treated with cross-sex hormones. Clin. Endocrinol..

[83] Lee H., McGovern K., Finkelstein J.S., Smith M.R. (2005). Changes in bone mineral density and body composition during initial and long-term gonadotropin-releasing hormone agonist treatment for prostate carcinoma. Cancer.

[84] Galvao D.A., Nosaka K., Taaffe D.R., Spry N., Kristjanson L.J., McGuigan M.R., Suzuki K., Yamaya K., Newton R.U. (2006). Resistance training and reduction of treatment side effects in prostate cancer patients. Med. Sci. Sports Exerc..

[85] Segal R.J., Reid R.D., Courneya K.S., Malone S.C., Parliament M.B., Scott C.G., Venner P.M., Quinney H.A., Jones L.W., D’Angelo M.E.S. (2003). Resistance exercise in men receiving androgen deprivation therapy for prostate cancer. J. Clin. Oncol..

[86] Fu X., Wang H., Hu P. (2015). Stem cell activation in skeletal muscle regeneration. Cell Mol. Life Sci..

[87] Bruusgaard J.C., Johansen I.B., Egner I.M., Rana Z.A., Gundersen K. (2010). Myonuclei acquired by overload exercise precede hypertrophy and are not lost on detraining. Proc. Natl. Acad. Sci. USA.

[88] Egner I.M., Bruusgaard J.C., Eftestøl E., Gundersen K. (2013). A cellular memory mechanism aids overload hypertrophy in muscle long after an episodic exposure to anabolic steroids. J. Physiol..

[89] Gundersen K. (2011). Excitation-transcription coupling in skeletal muscle: The molecular pathways of exercise. Biol. Rev. Camb. Philos. Soc..

[90] Gundersen K. (2016). Muscle memory and a new cellular model for muscle atrophy and hypertrophy. J. Exp. Biol..

[91] Staron R.S., Leonardi M.J., Karapondo D.L., Malicky E.S., Falkel J.E., Hagerman F.C., Hikida R.S. (1991). Strength and skeletal muscle adaptations in heavy-resistance-trained women after detraining and retraining. J. Appl. Physiol..

[92] Taaffe D., Marcus R. (1997). Dynamic muscle strength alterations to detraining and retraining in elderly men. Clin. Physiol..

[93] Neal A., Boldrin L., Morgan J.E. (2012). The satellite cell in male and female, developing and adult mouse muscle: Distinct stem cells for growth and regeneration. PLoS ONE.

[94] Seaborne R., Strauss J., Cocks M., Shepherd S., O’Brien T.D., Van Someren K.A., Bell P.G., Murgatroyd C., Morton J.P., Stewart C.E. (2018). Methylome of human skeletal muscle after acute & chronic resistance exercise training, detraining & retraining. Sci. Data.

[95] Seaborne R.A., Strauss J., Cocks M., Shepherd S., O’Brien T.D., Van Someren K.A., Bell P.G., Murgatroyd C., Morton J.P., Stewart C.E. (2018). Human Skeletal Muscle Possesses an Epigenetic Memory of Hypertrophy. Sci. Rep..

[96] Karunasena N., Han T.S., Mallappa A., Elman M., Merke D.P., Ross R.J., Daniel E. (2017). Androgens correlate with increased erythropoiesis in women with congenital adrenal hyperplasia. Clin. Endocrinol..

[97] Hicks B.M., Klil-Drori A., Yin H., Campeau L., Azoulay L. (2017). Androgen Deprivation Therapy and the Risk of Anemia in Men with Prostate Cancer. Epidemiology.

[98] Snyder P.J., Peachey H., Berlin J.A., Hannoush P., Haddad G., Dlewati A., Santanna J., Loh L., Lenrow D.A., Holmes J.H. (2000). Effects of testosterone replacement in hypogonadal men. J. Clin. Endocrinol. Metab..

